# Molecular Genetic Diversity of Major Indian Rice Cultivars over Decadal Periods

**DOI:** 10.1371/journal.pone.0066197

**Published:** 2013-06-21

**Authors:** Gangaprasad Choudhary, Nagireddy Ranjitkumar, Malathi Surapaneni, Dondapati Annekitty Deborah, Abhilash Vipparla, Ghanta Anuradha, Ebrahimali Abubacker Siddiq, Lakshminarayana Reddy Vemireddy

**Affiliations:** Institute of Biotechnology, Acharya NG Ranga Agricultural University, Rajendranagar, Hyderabad, Andhra Pradesh, India; University of Hyderabad, India

## Abstract

Genetic diversity in representative sets of high yielding varieties of rice released in India between 1970 and 2010 was studied at molecular level employing hypervariable microsatellite markers. Of 64 rice SSR primer pairs studied, 52 showed polymorphism, when screened in 100 rice genotypes. A total of 184 alleles was identified averaging 3.63 alleles per locus. Cluster analysis clearly grouped the 100 genotypes into their respective decadal periods i.e., 1970s, 1980s, 1990s and 2000s. The trend of diversity over the decadal periods estimated based on the number of alleles (*Na*), allelic richness (*Rs*), Nei’s genetic diversity index (*He*), observed heterozygosity (*Ho*) and polymorphism information content (PIC) revealed increase of diversity over the periods in year of releasewise and longevitywise classification of rice varieties. Analysis of molecular variance (AMOVA) suggested more variation in within the decadal periods than among the decades. Pairwise comparison of population differentiation (*Fst*) among decadal periods showed significant difference between all the pairs except a few. Analysis of trends of appearing and disappearing alleles over decadal periods showed an increase in the appearance of alleles and decrease in disappearance in both the categories of varieties. It was obvious from the present findings, that genetic diversity was progressively on the rise in the varieties released during the decadal periods, between 1970s and 2000s.

## Introduction

Ever since the domestication of crop plants, man has been improving them giving selection emphasis to traits that suit his agro-ecological and socioeconomic needs. In rice, like many crops, selection preference has been for improvement of yield enhancing traits like compact panicle with more grains/panicle, large seed size, non-shattering habit etc. The selection process continued for centuries result in cultivars far different from the wild/weedy progenitor species in their habit and potential. Since the advent of the short statured high yielding varieties in the mid-sixties, the selection priority of breeders has been for higher stability or performance, need based crop duration, tolerance to various stresses and consumer preferred grain quality. Excessive breeding emphasis in this direction given during the last 50 years knowingly or unknowingly has led to some sort of genetic uniformity among the currently cultivated high yielding varieties. The improved germplasm is being excessively depended on for needed variability for progressive improvement of the crop with no precise knowledge of extent of exploitable variability beyond simply inherited traits. Also it is not clear to what extent breeding strategies *in vogue* have been facilitating to broaden or narrow the genetic diversity in the breeding nurseries of rice.

Precise knowledge of exploitable genetic diversity and genetic relatedness among the constituents of the improved gene pool is vital for meticulously planning and executing target directed breeding especially when genetic relatedness among the improved parental sources appears to become increasingly narrow. Unlike morpho-physiological traits used earlier to estimate genetic variability/relatedness, molecular markers have become quite handy in precisely understanding the extent of genetic divergence among varieties being chosen these days as parental sources in breeding programs. Many recent scientific papers tracing the trend of genetic diversity in crop varieties released over the years reveal the diversity levels to often fluctuate strongly from one time period to the successive periods [1]–[3] and sometimes show conflicting results [4], [5]. By and large no clear pattern has emerged from such studies in the past, as both increasing and decreasing trends in diversity have been observed [6]–[8]. Diversity loss in rice during the last few centuries is obvious from the rapid decline of rice varieties from 400,000 before colonialism to 30,000 by mid-19th Century. This number has further come down during the era of the Green revolution as few high yielding varieties replaced thousands of native varieties [9]. Estimation of genetic diversity in varieties released in different crops during the last century reveals no clear trends [10]. In rice, both decreased [11] as well as increased trend of diversity [12] has been reported.

Since the introduction of semidwarf high yielding varieties in the mid 1960s, simultaneously launch of the All India Coordinated Rice Improvement Programmes (AICRIP) and active involvement of International Rice Research Institute (IRRI), Philippines facilitated rapid exchange of germplasm among breeders, institutes and between countries culminating in the release of around 1000 high yielding varieties in the country. Despite such large number of varieties developed using diverse germplasm, molecular marker based diversity analysis has shown the genetic base of Indian rice gene pool to be surprisingly narrow [13], [14]. Moreover, with regard to trends of genetic diversity in major Indian rice cultivars, however, little work has been done Recently, hypervariable microsatellite markers evenly distributed in rice genome have been demonstrated to be quite effective in estimating genetic diversity [15] Keeping the foregoing, the present study was undertaken to understand the level and trend of genetic diversity in the high yielding varieties developed in India over decadal periods between 1970s and 2000s employing hypervariable microsatellite markers.

## Materials and Methods

### Plant Material

The experimental material comprised of 100 rice cultivars, which included 89 high yielding varieties released between 1970 and 2010 in different states of India and 11 traditional varieties (Table 1 and Table S1). They were grouped according to the decade of their release (herefrom regarded as “year of releasewise”) for general cultivation across regions *viz*., 1970s, 1980s, 1990s and 2000s. They were as well classified into “longevitywise” as differences in genetic diversity could be observed between them, especially when a variety is popular within and across decades.

**Table 1:** Rice varieties used in the present study.

DecadalPeriod	Released Rice Varieties	Total No.
Landraces	INRC10192, Lalnakanda, Hasansona, Solumpiket, Basmati370, Acharmati, Bate Aus, Aus Boro, Dular, Aus Bako, Azucena	11
1970s	Manoharsali, Taichung Native-1, Jaya, Tella Hamsa, Rajeswari, Annapoorna, Jyothi, PR-106, N-22 (Nagina-22), Surekha (WL-13400),Prabhat (MTU-3626), Swarnadhan, Masur, WGL3200	14
1980s	Annada, Rasi, SasyaSree, PLA1100, Swarna (MTU-7029), Himalaya-2, Kalinga III, Vikas, CO-43, Keshari, Parijat, VL Dhan-16,VLDhan-206, Pathara, Prasanna, Sonasali, Pothana, Sabita, HKR-120, Mandya Vijaya, Suraksha, Satya, ASD-17, CSR-10, Kasturi,Kanak, Pusa Basmati-1, Tikkana (NLR-27999), Pinakini (NLR-9672-96), Samba Mahsuri (BPT-5204), Vanaprabha	30
1990s	Krishnaveni (MTU-2077), Haryana Basmati, IR-64, Chandana, VLDhan-221, Kavya (WGL-48684), Sneha, Swarnamukhi (NLR-145),Badami, Pusa-44, Ghanteswari, Khandagiri, Nilagiri, Himalaya-2216, Mahi Sugandha, Taraori Basmati, Basmati-386, KrishnaHamsa, Nidhi, VLDhan-61, Triguna, CSR-13, Lalithagiri, Uydyagiri	24
2000s	Bharani (NLR-30491), Srikakulam Sannalu, Somasila (NLR-33358), Sravani (NLR-33359), Cottondora Sannalu (MTU1010), PR-115,Mugad Sugandha-1, Sumathi, Vandana, Yamini (CSR 30), Pusa sugandha-3, Super, Pusa-1121, WGL-32100, MTU-1061 (Indra),Taramati, Suganda samba, Vasumati, Sharbathi, Improved Samba mahsuri and Swarna sub1	21

### DNA Extraction

Genomic DNA was isolated from 20 day-old seedlings germinated in sterile petri dishes lined with moist filter paper using the CTAB (Cetyl Try methyl Ammonim Bromide) method described [16] with some modifications. The purity and concentration of the isolated genomic DNA samples were estimated by UV-absorption spectrophotometer (Beckman DU 650 model) as per the procedure described by Sambrook [17]. Quantification of DNA was done by analyzing the purified DNA on 0.8% agarose gel with lambda (λ) *Hind* III DNA as standard. Based on the intensity and thickness of genomic DNA bands, as compared to lambda (λ) *Hind* III DNA, the concentration and quality of DNA in individual samples were determined.

### SSR Marker Analysis

In all, 64 hypervariable microsatellite markers distributed on all the 12 chromosomes of rice were picked up from our previous study [15]. These markers covering 236.8 Mb of physical distance with average distance between them being 5.92 Mb. PCR reactions were carried out in 10 µl reaction volume containing of 10× PCR buffer (10 mM Tris-HCl pH 8.3, 50 mM KCl), 1.5 mM MgCl_2_, 0.2 mM each dNTPs, 5 pmol of each forward and reverse primer, 0.5U of Taq DNA polymerase (NEB), and 5 ng of genomic DNA. Reactions were carried out in GenAmp PCR system 9700 (Applied Biosystems, USA) thermal cycler using the following temperature profile: an initial denaturation of 5 min at 94°C followed by 35 cycles of 45 s at 94°C, 45 s at 55°C and 1 min at 72°C, then a final extension of 5 min at 72°C. Amplification products were resolved on 3% metaphor agarose gels using a horizontal gel electrophoresis unit (CBS Scientific, USA). The DNA fragments were then visualized under UV-transilluminator and documented using ALPHA IMAGER gel documentation system (Alpha Innotech, USA) which was stored for further scoring and permanent records.

### Data Analysis

Only clear and unambiguous bands of SSR markers were scored. The sizes of the amplified fragments were estimated with the help of Alpha image software by Gel documentation system using 100 bp DNA ladder (NEB) as size standard. Markers were scored for the presence (1) or absence (0) of the corresponding band among the genotypes. To measure the informativeness of the markers, polymorphism information content (PIC) for each of the SSR markers was computed according to the formula: PIC = 1-ΣPi^2^ - ΣΣPi^2^ Pj^2^ where ‘i’ is the total number of alleles detected for SSR marker and ‘Pi’ is the frequency of the i^th^ allele in the set of hundred genotypes investigated and j = i+1 [18].

Genetic diversity parameters *viz.,* number of alleles *(Na)*, observed heterozygosity (*Ho*), Shannon Index (*I*) and Nei’s genetic diversity index (*He*) [19] were evaluated using POPGENE v 1.31 (http://www.ualberta.ca/~fyeh ). The allelic richness (*Rs*), is a measure of the number of alleles independent of the sample size, was measured using FSTAT [20]. Significant difference in genetic diversity parameters between each pair of the decadal periods was calculated using the Wilcoxon matched pairs test, a nonparametric alternative to the *t*-test (http://www.stattools.net/Wilcoxon_Pgm.php). In addition, number of rare alleles (number of alleles in less than 5% of the population) and high frequency or common alleles (number of alleles in more than 85% of the population) were estimated using Microsoft Excel 2007. The *F*-statistics (*Fst*) were used to analyze genetic differentiation of the varieties in all possible pairs of decades based on allelic discrepancy at each locus by the procedure of AMOVA (Analysis of Molecular Variance), besides the genetic variation within and among the populations using the software ARLEQUIN v 2.0 [21]. The appearance and disappearance of alleles and number of private alleles have been estimated using the CONVERT v 1.31software [22]. In order to overcome the problem of unequal sample size, resampling was done using an in-house script for R software [23]. The UNJ (Unweighted Neighbour Joining method) cluster analysis followed by bootstrap analysis with1000 permutations for total cultivars was carried out using DARwin 5.0.145 (http://darwin.cirad.fr/). The dendrogram based on unbiased genetic distances among decadal periods was constructed by UPGMA (Unweighted pair-group method with arithmetic average) employing POPGENE v 1.31.

## Results and Discussion

### Hypervariable Microsatellite Marker Analysis

**Figure 1:**
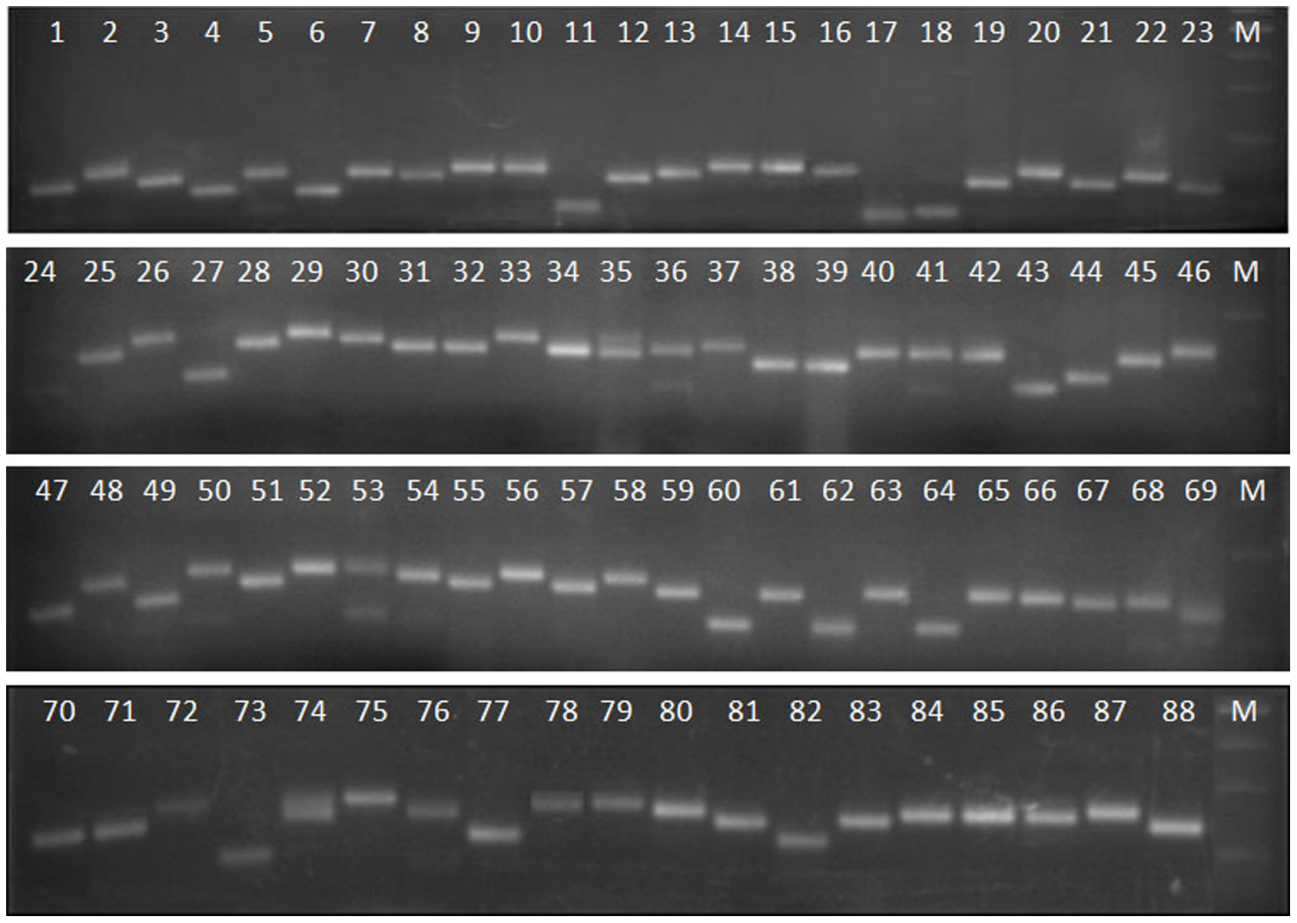
A representative gel picture of screening of rice varieties with RM562. For decoding of the numbers refer Table S1.

**Figure 2:**
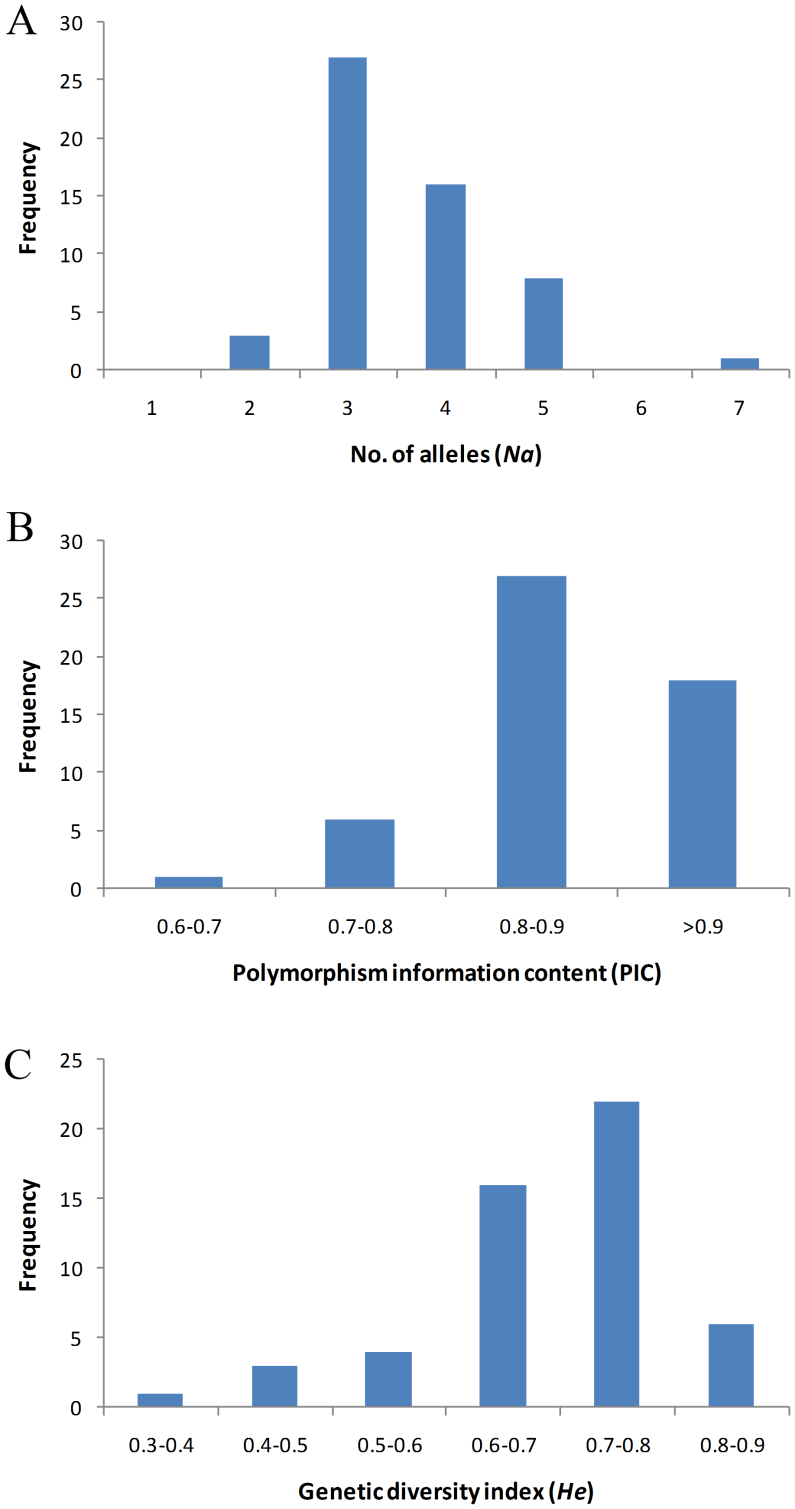
Distribution of number of alleles (*Na*) (A), polymorphism information content (PIC) (B) and genetic diversity (*He*) (C) estimated from 100 rice varieties using 52 hypervariable microsatellite loci.

Sixty four hvRM (hypervariable rice microsatellite) markers distributed evenly on all the 12 chromosomes were chosen to assess the genetic relatedness among the 100 genotypes. Fifty-two of them were found to show polymorphism (81.25%)(Table 2 and Figure 1). In all, 184 alleles were identified by amplification of the 52 polymorphic hvRMs with an average number of alleles of 3.6 per locus, with the number ranging from 3 (RM16416) to 7 (RM8207) (Table 2). This was significantly lower than the average number of alleles reported by Jain *et al* (7.8) [24], Spada *et al.* (7.2) [25] and Zhu *et al.* (4.37) [26] and higher than the Chuan-Guang and Gui-Quan [27]. Yu *et al.* [28] studied 193 rice accessions drawn from 26 countries using 101 SSR primer pairs and detected an average allele number of 6.3 per locus, which is also higher than the value reported here. Luce *et al.* [29] analyzed 419 rice accessions from the gene banks in five European countries using 16 SSR loci (different from the ones we selected) and reported an average of 9.1 alleles per locus. The higher value than the present study (3.6) could probably be as a result of larger number of accessions used by these authors. Of the 52 loci, 27 were with three alleles while 16 loci with four alleles (Figure 2). All the 52 markers revealed high PIC values the range being between 0.67 (RM16416) and 0.97 (RM14735) with more than 50% loci in the range of 0.8 to 0.9 (Figure 2). Some of the hvRM markers like RM11340, RM12548, RM13584, RM14270, RM14735, RM15580, and RM22273 showed high PIC values in our previous study as well [15]. The average PIC value estimated in the present study (0.87) is more than that of the previous studies by Giarrocco *et al.* [30] (0.69) and Jayamani *et al.* [31] (0.67) possibly because of hypervariable microsatellite markers used. Recently, it has been demonstrated that microsatellite markers of hypervariable nature would be more polymorphic than non-hypervariable markers [15] The *He* values ranged from 0.15 (RM12031) to 0.76 (RM14735) with an average of 0.59 and more than 40% of the loci were in the frequency of 0.7 to 0.8 (Figure 2). The average *He* value of the present study is slightly lower than the earlier studies [11], [32] due to the inclusion of only major Indian rice cultivars, which largely belong to *indica* sub group.

**Table 2:** Molecular diversity parameters of the microsatellite markers used in the study.

Locus	Chr.	SSR motif	*Na*	*Rs*	PIC	*Ho*	*He*	*I*
RM562	1	(AAG)13	5	1.63	0.94	0.04	0.73	1.34
RM11313	1	(AAT)23	3	1.49	0.86	0.00	0.56	0.90
RM11340	1	(AT)42	4	1.47	0.89	0.07	0.50	0.89
RM11356	1	(AT)31	3	1.54	0.88	0.12	0.56	0.89
RM11597	1	(AT)42	3	1.48	0.9	0.00	0.57	0.92
RM12031	1	(AG)40	3	1.18	0.74	0.02	0.15	0.32
RM12253	1	(AAT)23	4	1.57	0.91	0.12	0.60	1.02
RM12292	1	(AT)38	3	1.62	0.92	0.14	0.66	1.09
RM12353	2	(AAT)32	4	1.40	0.86	0.16	0.56	0.91
RM12548	2	(AT)46	3	1.57	0.9	0.01	0.60	0.99
RM13131	2	(AT)32	3	1.44	0.91	0.01	0.55	0.90
RM13584	2	(AATC)5	3	1.57	0.88	0.05	0.63	1.05
RM14270	3	(AT)46	4	1.60	0.93	0.00	0.66	1.09
RM14735	3	(AT)42	5	1.60	0.98	0.06	0.76	1.52
RM14778	3	(AT)37	3	1.62	0.89	0.01	0.63	1.04
RM15004	3	(AAT)38	4	1.64	0.91	0.05	0.65	1.14
RM15580	3	(AT)50	3	1.56	0.85	0.02	0.59	0.95
RM16416	4	(AAT)12	3	1.32	0.69	0.02	0.34	0.53
RM16577	4	(AT)29	4	1.60	0.91	0.13	0.62	1.03
RM17405	4	(AAT)36	3	1.47	0.88	0.00	0.49	0.78
RM17669	4	(AT)30	3	1.58	0.9	0.03	0.58	0.95
RM5693	5	(AAT)18	4	1.59	0.78	0.59	0.61	1.08
RM5844	5	(AAT)20	5	1.68	0.94	0.01	0.68	1.24
RM5907	5	(AAT)19	3	1.50	0.86	0.01	0.48	0.84
RM18384	5	(AAG)22	3	1.42	0.9	0.26	0.40	0.80
RM18639	5	(AAT)17	5	1.66	0.78	0.81	0.68	1.26
RM19545	6	(AAT)21	4	1.58	0.91	0.00	0.58	0.94
RM20037	6	(AT)38	4	1.60	0.92	0.01	0.63	1.05
RM20710	6	(AT)46	5	1.70	0.97	0.49	0.76	1.52
RM21693	7	(AT)44	5	1.60	0.93	0.05	0.66	1.19
RM21941	7	(AAT)25	3	1.56	0.84	0.05	0.58	0.98
RM6965	7	(AAG)15	3	1.49	0.91	0.03	0.54	0.90
RM22250	8	(AAT)30	5	1.71	0.94	0.05	0.71	1.34
RM22544	8	(AAT)20	4	1.69	0.89	0.41	0.73	1.35
RM22565	8	(ACAT)15	4	1.67	0.9	0.08	0.68	1.26
RM22688	8	(AAT)28	3	1.34	0.74	0.01	0.38	0.56
RM22273	8	(AT)35	4	1.66	0.83	0.34	0.68	1.24
RM23017	8	(AAT)18	3	1.42	0.83	0.05	0.51	0.79
RM23036	8	(AGAT)15	4	1.62	0.9	0.18	0.64	1.11
RM23362	8	(AAG)19	5	1.60	0.94	0.46	0.66	1.22
RM23741	9	(AAT)28	4	1.59	0.78	0.85	0.59	0.99
RM24015	9	(AGAT)9	4	1.68	0.86	0.68	0.70	1.29
RM24044	9	(AAG)11	3	1.45	0.88	0.19	0.50	0.84
RM24260	9	(AAT)31	3	1.55	0.88	0.00	0.63	1.04
RM5708	10	(AAT)22	4	1.68	0.83	0.47	0.69	1.30
RM8207	10	(AAG)23	7	1.69	0.95	0.05	0.69	1.34
RM25262	10	(AAT)38	3	1.41	0.79	0.08	0.47	0.66
RM25969	11	(AAG)18	3	1.34	0.81	0.01	0.40	0.72
RM26190	11	(AGAT)13	3	1.47	0.92	0.01	0.59	0.96
RM26632	11	(AAAG)9	3	1.53	0.85	0.01	0.57	0.95
RM27840	12	(AAT)37	3	1.54	0.86	0.02	0.58	0.95
RM28279	12	(AATC)8	3	1.56	0.84	0.28	0.61	1.01
Mean			3.69	1.55	0.87	0.15	0.59	1.02
SD			0.90	0.11	0.06	0.22	0.11	0.24

Chr.- Chromosome; *Na*-Number of alleles; PIC-Polymorphism Information Content; *Rs*: Allelic richness; *Ho*- Observed heterozygosity; *He*- Nei’s genetic diversity; *I*- Shannon Index; SD-Standard Deviation.

### Overall Genetic Diversity in Popular Varieties of Rice

**Figure 3:**
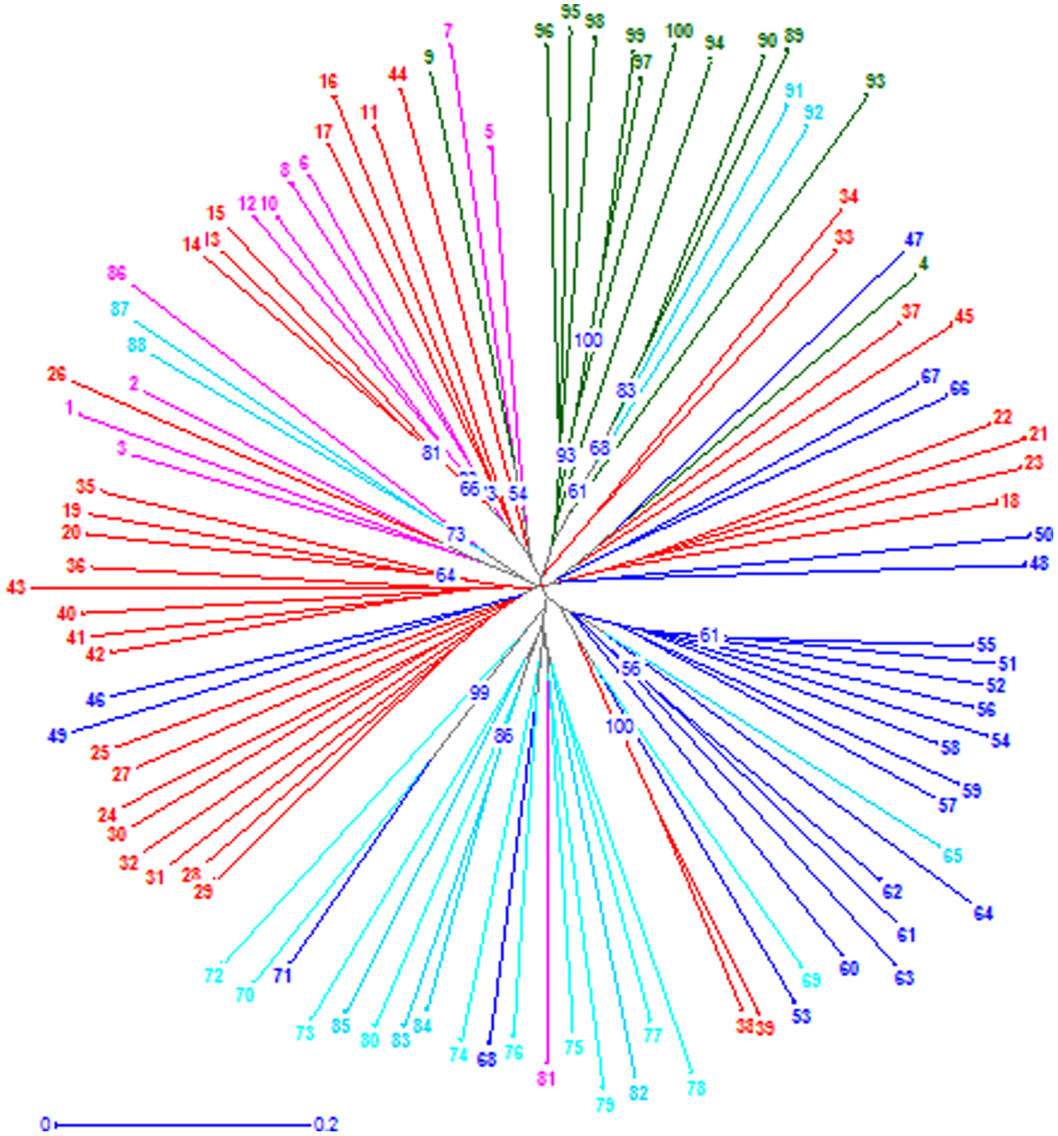
Genetic relationship among 100 rice varieties estimated using Unbiased Neighbour-Joining dendrogram of 52 hypervariable microsatellite loci. Landraces- green colored;1970s-pink colored;1980-red colored;1990s-dark blue colored;2000s-sky blue colored. Rice varieties were represented in numbers. For full details of the varieties refer Table S1.

The unweighted neighbour-joining (UNJ) dendrogram constructed on the basis of genetic similarity matrix grouped the 100 genotypes into five clusters *viz.,* landraces, 1970s, 1980s, 1990s, and 2000s (Figure 3). The phylogeny tree reveals that appearing of some of the varieties released during one decade in another decade due to the presence of common parents in their pedigree. For instance, ASD17 and CSR10 belonging to the decadal period of the 1980s clustered with those of the 1970s as IR8 and Jaya of the 1970s being common in their parentage. Varieties of the decadal periods1990s and 2000s comprised largely of varieties released during their respective decades. However, varieties like Tikkana and Pinakini of the 1980s and Somasila and Srikakulam Sannalu of 2000s have been found to cluster with those of the 1990s. Interestingly, Basmati varieties irrespective of their year of release formed a separate sub-cluster. This result is in agreement with previous reports by Glaszman [33], Nagaraju *et al* [34] and Narshimulu *et al* [15]. Equally and interestingly the recently released varieties developed through marker-assisted breeding *viz.,* Swarna-sub1 and improved Samba mahsuri grouped with landrace cluster instead of the expected grouping with the 2000s decade where in their original parents *viz.,* Swarna and Samba mahsuri exist. This could be due to the presence of part of the donor genomes of FR13A, a flood resistant landrace the source for *sub-1* gene and SS1113, the source of bacterial blight resistance genes (*xa13*, *xa5* and *Xa21*) in improved Samba mahsuri, remaining in the improved versions of the varieties even after many backcrosses. Longevitywise clustering of the 100 genotypes too was in total agreement with the decadewise clustering. Even though some varieties were popular beyond the decade(s) of their release, they do not affect the clustering pattern of the genotypes as well as the genetic diversity of successive decadal periods. For instance, Tella Hamsa and Jyoti remain popular even as late as in the 2000s, albeit released decades back in the states of Andhra Pradesh and Kerala, respectively.

**Figure 4:**
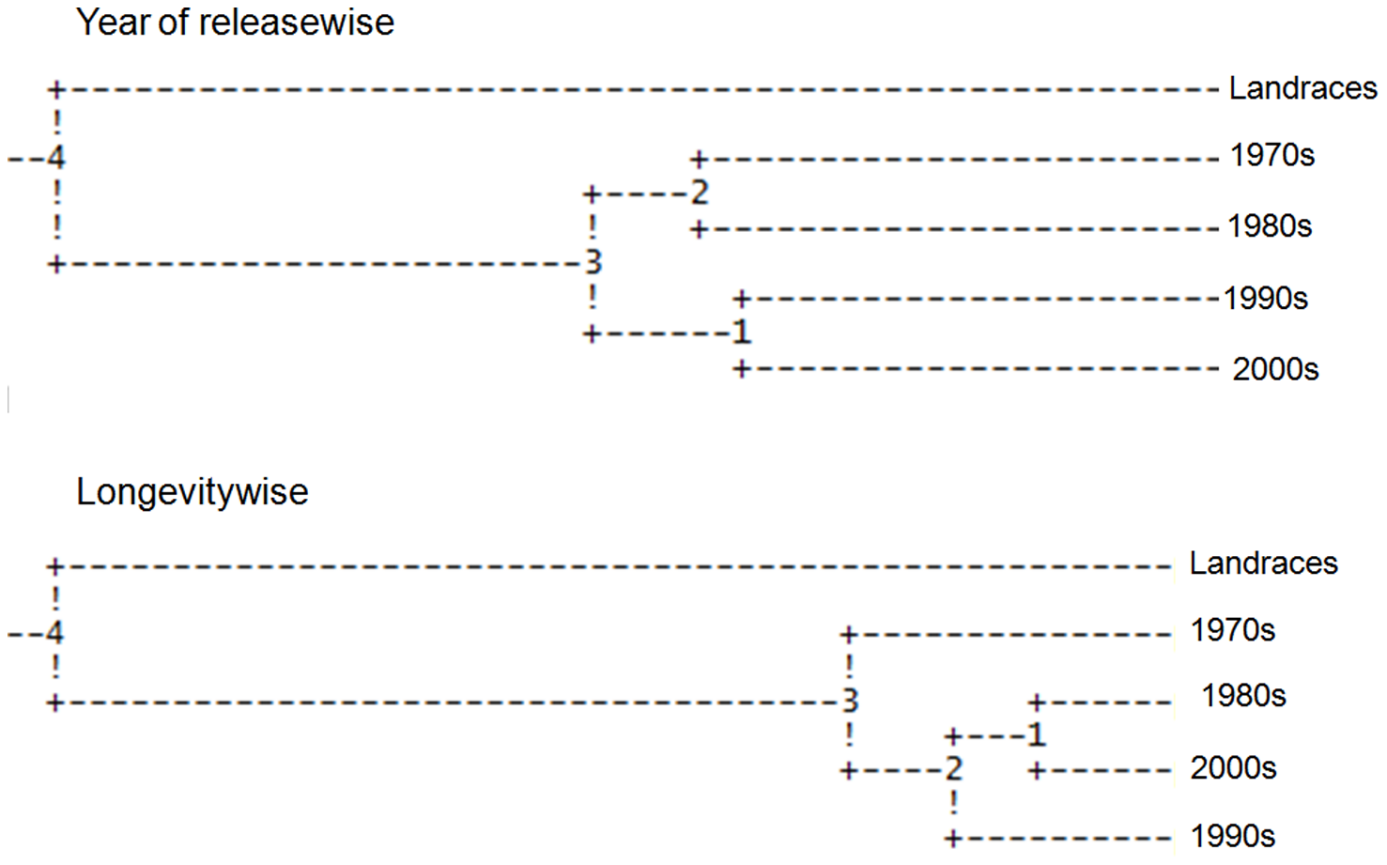
UPGMA dendrogram based on Nei’s genetic distances using POPGENE v 1.31 showing the genetic relationship of rice genotypes among decadal periods. The genetic distances between different groups are as follows. Year of releasewise: 4 and Landraces - 10.96, 4 and 3–5.01, 3 and 2–0.97, 2 and 1970s - 4.97, 2 and 1980s- 4.97, 3 and 1–1.41, 1 and 1990s-4.52 and 1 and 2000s-4.52. Longevitywise: 4 and Landraces −9.55, 4 and 3–6.76, 3 and 1970s - 2.78, 3 and 2 -0.93, 2 and 1 - 0.66, 1 and 1980s - 1.18, 1 and 2000s - 1.18, 1 and 1990s - 1.85.

Genetic distance between each pair of decadal periods suggest that it was the highest between the landraces and the high yielding varieties of the 1990s (0.2715), while the lowest between the decades 1990s and 2000s (0.0905) in the year of releasewise group. In case of longevitywise group also genetic distance was highest between the landraces and the high yielding varieties of the 1990s (0.210) but lowest between those of the 1980s and 2000s (0.0237) (Table 3). A dendrogram constructed based on Nei’s genetic distance also confirms the above findings as the genetic distance between landrace cultivars and the improved varieties released in 1990s being the highest (Figure 4).

**Table 3:** Nei’s Unbiased measures of genetic distance among decadal periods of major Indian rice cultivars.

Decade	Landraces	1970s	1980s	1990s	2000s
Landraces	****	0.204	0.182	0.210	0.169
1970s	0.206	****	0.052	0.059	0.056
1980s	0.189	0.099	****	0.046	0.024
1990s	0.272	0.126	0.118	****	0.029
2000s	0.211	0.132	0.100	0.091	****

Lower diagonal: Year of releasewise; Upper diagonal: Longevitywise.

### Trends of Genetic Diversity within Decadal Period

**Figure 5:**
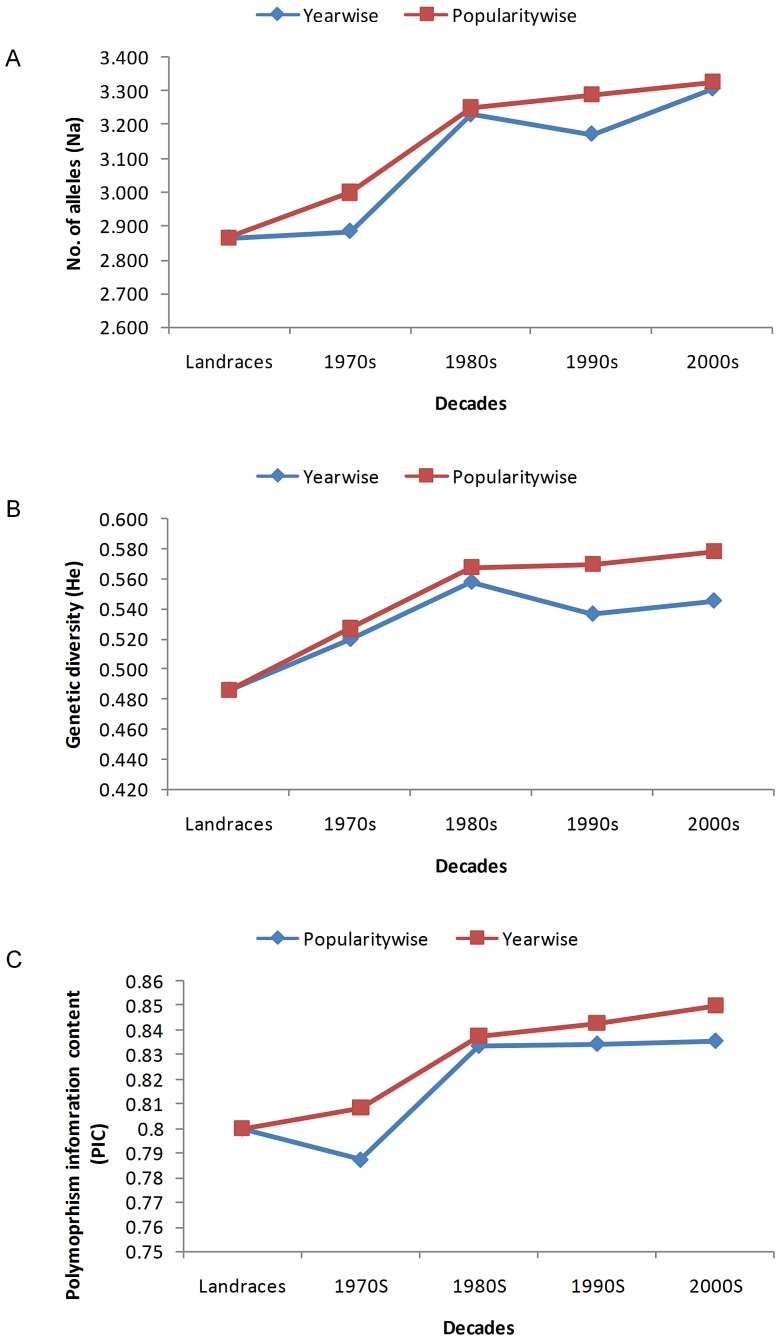
Changes in number of alleles (*Na*), Nei’s genetic diversity (*He*) and PIC values over decadal periods.

Genetic diversity trend of varietal groups released during different decades since the release of semi-dwarf high yielding varieties (1970s to 2000s) along with sets of landraces and Basmati accessions was studied based on number of alleles *(Na)*, Nei’s genetic diversity index (*He*) and polymorphism information content (PIC) (Figure 5 and Table S2). As well, the diversity within decadal periods was estimated through separate analysis of year of releasewise as well as longevitywise varieties. The diversity trends as estimated through a set of parameters are as under:

Overall increasing trend of number of alleles (*Na)* was observed from landraces to varieties of 2000s in the categories of both year of releasewise and longevitywise classified rice varieties. *Na* values were increased by 13.38% and 13.87% from landraces to 2000s while it was 12.79% and 9.83% from 1970s to the 2000s, respectively for both the categories. On an average, landraces to total modern cultivars percentage of *Na* increase has been observed to be 9.02 for year of releasewise and 10.91 for longevitywise varieties. This is in contrast to the observation of Wei *et al.* [11], who reported decreasing trend of the *Na* score of 7.8% from the 1950s to the 1990s.

Genetic diversity index (*He*) also reveals that the genetic diversity has been increased approximately 10% from landraces to 2000s and landraces to modern cultivars in the year of releasewise category while it was 16.02% and 13.39% for longevitywise category, respectively. However, from 1970s to 2000s the trend of increase was 4.71% and 8.92% for year of releasewise and longevitywise varieties, respectively. The observed trends in the present study are in agreement with Mantegazza *et al.,* [12], who have reported increase of genetic diversity of Italian rice cultivars between 1850 and 2001. The same trend has been reported also by Morin *et al.,* [35]. However, these findings vary with the report of Wei *et al.,* [11] who observed 7% loss of genetic diversity between 1950s and 1990s.

Polymorphism information content (PIC) also shows increasing trend over decadal periods, though not to the level of *Na* and *He*. The percent increase of PIC values from landraces to all modern cultivars, from landraces to 2000s and from 1970s to 2000s as estimated by year of releasewise were 2.75, 4.25 and 5.77%, respectively, while the corresponding values estimated longevitywise are 4.17, 5.88 and 4.87%. Overall, the trends as measured by the parameters of *Na*, *He* and PIC appear to slightly slow down in the decadal period of 1990s but regains its increasing trend in that of 2000s.

The genetic diversity in Indian cultivars (*He* = 0.54) as estimated by the Nei’s genetic diversity index in the present study is nearly equal to that of the representative world collection of *indica* varieties (*He* = 0.55). It is, however, overwhelmingly high as compared to the representative world collection of temperate *japonica* (*He* = 0.39) [36]. Thus, the findings of the present study confirms further, the *indica* varietal group to be much more genetically diverse than *japonica* group which is in agreement with many earlier reports [32], [37].

**Figure 6:**
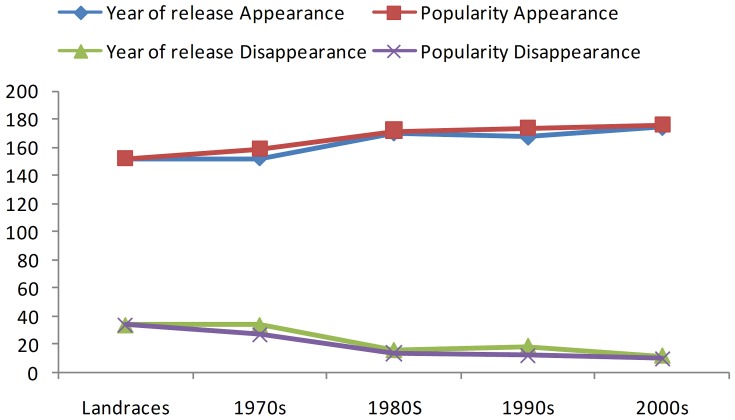
Appearance and disappearance of alleles over decadal periods.

Overall, the present investigation reveals a genetic diversity as estimated using different parameters such as allele number (*Na*), polymorphism information content (PIC) and genetic diversity index (*He*), to increase progressively in the advancing decadal periods from 1970s to 2000s and interestingly, it was higher as compared to even landraces. Use of genotypes, which cover longer periods always offer added advantage in temporal studies. In the present study only a few landraces which are being cultivated for their certain features by local farmers has been included to compare genetic diversity in varieties released before and after 1970s. The analysis of genetic diversity using various parameters clearly reveals the increasing trend from landraces to the decadal period 2000s. While this observation is in agreement with those of Mantegazza *et al* [12], who have reported steady increase in the levels of gene diversity in Italian rice germplasm, it was not so with the findings of Steele *et al* [38] who have reported no change in the level of diversity following the introduction of modern rice varieties. Wei *et al* [11] and Yuan *et al* [32] report, however, loss of more alleles in modern cultivars as compared to varieties released in the 1950s. Put together, the present findings and previous reports, it is reasonable to assume that global rice genetic diversity tend to increase over the decades by gaining as well as losing to different proportion of alleles. In the absence of phenotypic expression of alleles gained or lost in rice genome it would be difficult to conclude if they are of adaptive or agronomic value. Qualitative study of variation in gene diversity, over the decadal periods, as estimated by the total number of alleles that appeared and disappeared showed an increase in the appearance and decrease in the disappearance of alleles from landraces to 2000s in both the year of releasewise and longevitywise analysis (Figure 6). Though we presume that disappearance of certain alleles might be of deleterious nature and that appearance of new alleles to be of positive breeding value, it need not be so always. Thus, there is a need for still more precisely planned further study. As all the major cultivars of India are belonging to the *indica* subspecies of *Oryza sativa,* we could not compare with the *japonica* varieties in terms of allele appearance and disappearance. However, earlier [11] it was proved that more alleles loss has been observed in *indica* varieties than in *japonica.* This phenomenon can be attributed to the higher diversity nature of *indica* varieties compared to *japonica*.

Many reasons may be attributed to the progressively increased genetic diversity over the decadal periods between 1970s and 2000s. In the initial decades, the advent of semidwarf plant type based exotic high yielding varieties like Taichung (Native)-1 and IR8 and varieties derived from crosses involving them with traditional Indian varieties could have introduced large variability for yield, earliness, photo-insensitivity, seed non-dormancy etc. In the subsequent decades, the focus has been shifted towards the development of polygenic durable resistance to multiple pathogens, adaptation to diverse rice ecologies and consumer preference driven quality improvement. For instance, high yielding varieties of the initial decades were more susceptible to indigenous as well as introduced diseases and insect pests like bacterial blight, tungro virus, hoppers etc. warranting to exploit exotic cultivar and wild/weedy sources. Convergent breeding efforts have made most of the high yielding varieties of today multiple pest resistant. Given the challenges ahead which have all the potential to impede further progress in crop improvement, there is need to enrich the variability employing multiparent breeding and exploitation of hidden variability from progenitor species and primitive cultivars.

### Trends of Genetic Diversity among Decadal Periods

Analysis of molecular variance (AMOVA) has revealed most of the variation was existed within decadal periods i.e., 92.12% and 96.66%, respectively for year of releasewise and longevitywise as against very low variation between the decadal periods i.e., 3.34% for longevitywise classified varieties and 7.88% for year of releasewise classification (Table 4). These results are in agreement with Yuan *et al.* [32]. Pairwise comparison of population differentiation (*Fst)* among decadal periods reveals significant genetic differentiation among all except between the landraces and the decadal period of 1970s as estimated by both year of releasewise and longevitywise analysis. Also there was no significant differentiation between the 1970s and rest of the decades 1980s to 2000s in longevitywise analysis (Table 5).

**Table 4:** Analysis of molecular variance (AMOVA) of major Indian rice cultivars.

Sample	Source of variation	df	Variance components	Percentage of variation
**Year of releasewise**	Among populations	4	0.549**	7.88
	Within populations	195	6.426***	92.12
	Total	199	0	
**Popularitywise**	Among populations	4	0.231**	3.34
	Within populations	287	6.690***	96.66
	Total	291	0	

df: degrees of freedom; p>0.001-*** : p>0.01-**.

**Table 5:** Pairwise population differentiation (*Fst*) of major Indian rice cultivars among decadal periods.

Decade	Landraces	1970s	1980s	1990s	2000s
**Landraces**	0.00000	0.06822	0.05477***	0.08557***	0.05925***
**1970s**	0.05112	0.00000	0.0138	0.02299	0.02338
**1980s**	0.03997*	0.03957	0.00000	0.03998***	0.01241
**1990s**	0.13083***	0.07904***	0.10569***	0.00000	0.02389**
**2000s**	0.09004***	0.07927**	0.07507***	0.0665***	0.00000

Lower diagonal: Year of releasewise; Upper diagonal: Longevitywise.

p>0.001-***; p>0.01-**;p>0.1-*.

Locus-by-locus AMOVA showed significant (p<0.01) differences in genetic variation among the decadal periods as exhibited by 14 loci *viz*., RM11356, RM12031, RM13131, RM14735, RM15004, RM15580, RM5693, RM5907, RM18384, RM22554, RM22565, RM22688, RM23741, and RM8207. Interestingly, three loci viz., RM11313, RM12353, and RM6965 have been found to explain more than 20% of the variation among the decades (Table S3).

### Analysis of Population Specific Alleles

Analysis for identification of unique population specific alleles has revealed all the decadal periods found to show specific alleles with the exception of the 1970s as estimated by year of releasewise as well as longevitywise classification. As expected, the landraces exhibited as many as six (RM12031, RM15004, RM5844, RM21693, RM22250 and RM23362) and seven (RM12031, RM15004, RM5844, RM21693, RM22250, RM23362 and RM8207) specific alleles compared to the decadal periods in both year of rleasewise and longevitywise categories, respectively (Table S4). While the decadal periods 1980s (RM18384) and 1990s (RM5708) having one specific allele each, the period 2000s is with two (RM5844 and RM23741) specific alleles. Of the 10 population specific alleles identified in all, five are located in genic regions (RM5844, RM18384, RM22250, RM23362 and RM23741) while rest in non-genic regions. Seven of the 10 alleles specific to populations are comprised of tri-nucleotide repeats, especially of AAT repeat motif.

Qualitative analysis of genetic diversity revealed that the landraces were comprising of more number of population specific alleles as compared to the modern high yielding varieties suggesting loss of some of the landrace specific alleles on account of intensive human selection over the decades. The present findings are in agreement with those of earlier workers [11], [32] who have also reported loss of alleles in the progressively improved modern varieties. The reason can be attributed to conscious and simultaneous selection against alleles of adaptive value characteristic to landraces resulting in less number of certain alleles and selection for alleles of agronomic value in the modern cultivars.

### Effect of Genic-SSRs, Non-genic-SSRs and Gene-specific Markers on Genetic Diversity Trends

Ever since the domestication of rice, man’s breeding priority has been yield enhancement and “breeding out” the wild progenitor traits like lax panicle, shattering, awns, etc., and later the focus had been shifted towards “breeding in” the traits of plant architecture and resistance to biotic and abiotic stresses and acceptable cooking quality. In that long breeding process, except the genomic regions, which govern domestication related traits, a major part of the rice genome remains unchanged and hence large unfolded genetic diversity. To estimate genetic variability in crop improvement research, increasingly crop specific microsatellite markers are used. Since most of the microsatellites are non-coding, estimates made and inferences drawn based on them may not reflect true genomic diversity of a crop. To understand precisely the changing trends of genetic diversity in breeding material over periods, it is necessary to consider markers located both in genic and non-genic regions. In the present study, thus, microsatellite markers located in both genic and non-genic regions have been chosen to understand the level and trend of genetic diversity in varieties developed over the last four decades in comparison to the traditional varieties. The study using two marker types separately and together interestingly showed an increasing trend of genetic diversity (Figure S1, Table S5, Table S6). Analysis of gene specific marker data obtained from earlier studies also [39]–[41] clearly suggests an increasing trend of genetic diversity in Indian rice varieties developed over a period of four decades. In contrast, Qi *et* *al* [42] have reported initially declining trend of diversity in varieties developed between 1950 and 1980s and subsequently increasing trend as estimated based on both SSR markers and phenotypic traits in Chinese rice varieties.

### Analysis of Genetic Diversity Based on Region, Ecology, Grain Size and Days to 50% Flowering Periodwise

When genetic diversity parameters viz., number of alleles (*Na*), number of effective alleles (*Ne*), Shannon index (*I*) and Nei’s genetic diversity index (*He*) estimated according to their region of release in India (South, North and East), ecology (irrigated and rainfed), grain size (long and medium) and days to 50% flowering (early, medium early, medium and late) of a representative set of Indian rice varieties, observed variation was not correlating with their classification (Figure S2, Table S7). The reasons could be attributed to the repeated use of founder varieties such as T(N)1 and IR8 as well as their derivatives like Jaya, Rajeswari, Rasi, Prabhat, PR106 and Suraksha in the ancestry of many of the subsequently evolved varieties irrespective of their trait classification. Further, observation of pedigree records of some of the emerging new varieties released during the past two decades revealed that they were having some of the quality rice varieties such as Basmati370, Sona and BPT5204 (Samba mahsuri) as their donors but not the original founder varieties or their derivatives. The reason for this trend could be assumed as the changing consumer preferences towards quality rices. Keeping this trend in view breeders also being used quality rice varieties as the donors in their breeding programs.

### Conclusions

Despite the perception that genetic variability has declined over the decades in the improved rice gene pool the present findings and previous reports suggest genetic diversity in the improved cultivars has been increasing over the decades, while losing some of the alleles which could be of unimportant and not relevant to the changing breeding objectives. However, it is important to understand the kind of alleles being gained and lost over the decades so as to plan our future breeding/selection strategies for directed improvement of the crop. The study has overall reveals that adequate diversity still exists in the advanced cultivar gene pool for steady varietal improvement. In the diversity conservation exercise being practiced at national and global levels, therefore, much emphasis is to be given to improved germplasm as that of the customarily conserved gene pool comprising wild/weedy species and landraces so that the genes of importance are conserved and profitably utilized to meet future challenges. It is, however, important to monitor periodically the level and trend of genetic diversity while pursuing aggressively rice breeding research.

## Supporting Information

Figure S1 Changes in number of alleles *(Na)*, genetic diversity (*He*) over decadal periods using genic and non-genic SSRs along with gene-specific markers(previous studies).
(TIF)

Figure S2 Number of alleles *(Na)*, number of effective alleles (*Ne*), Shannon index (*I*) and Nei’s genetic diversity (*He*) estimated in different groups of rice varieties i.e., region, ecology, grain size and days to 50% floweringwise.
(TIF)

Table S1 Details of the rice cultiavars used in the study.
(XLS)

Table S2 Summary statistics of genetic diversity parameters of major Indian rice cultivars.
(DOCX)

Table S3 Locus by locus AMOVA among populations.
(DOCX)

Table S4 Population specific alleles and their corresponding markers.
(DOC)

Table S5 List of genic and non-genic SSRs and their functions.
(DOC)

Table S6 Summary statistics of genetic diversity parameters of Indian rice cultivars using different microsatellite classes.
(DOCX)

Table S7 Summary statistics for genetic diversity parameters of Indian rice varieties classified according to their region, ecology, grain size and days to 50% flowering.
(DOCX)
