# Supplementary figures and images for: Molecular Genetic Diversity of Major Indian Rice Cultivars over Decadal Periods
Source: PLoS One. 2013 Jun 21;8(6):e66197. doi: 10.1371/journal.pone.0066197 (PMC3689748; doi:10.1371/journal.pone.0066197)

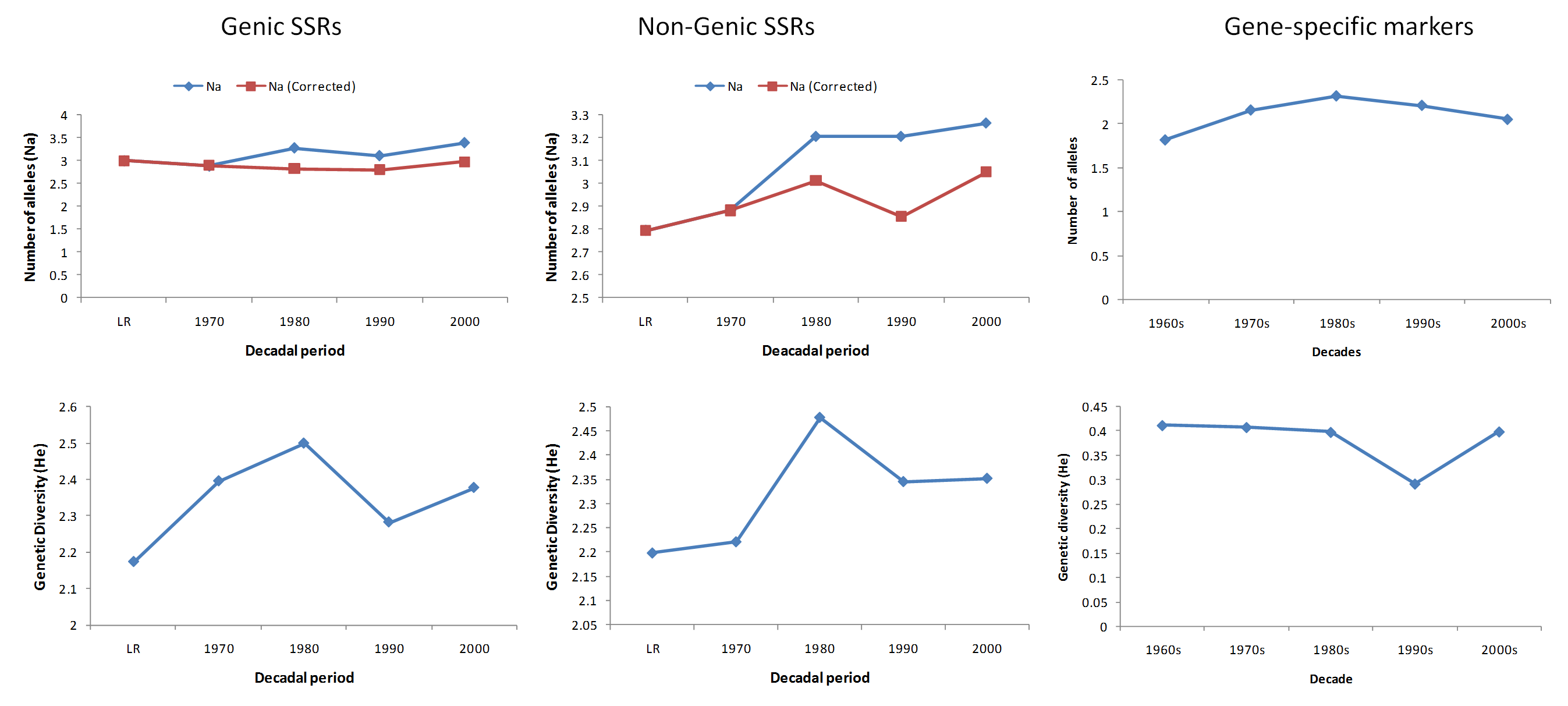

Supplement: Figure S1 — Changes in number of alleles (Na), genetic diversity (He) over decadal periods using genic and non-genic SSRs along with gene-specific markers(previous studies). (TIF) [file pone.0066197.s001.tif]

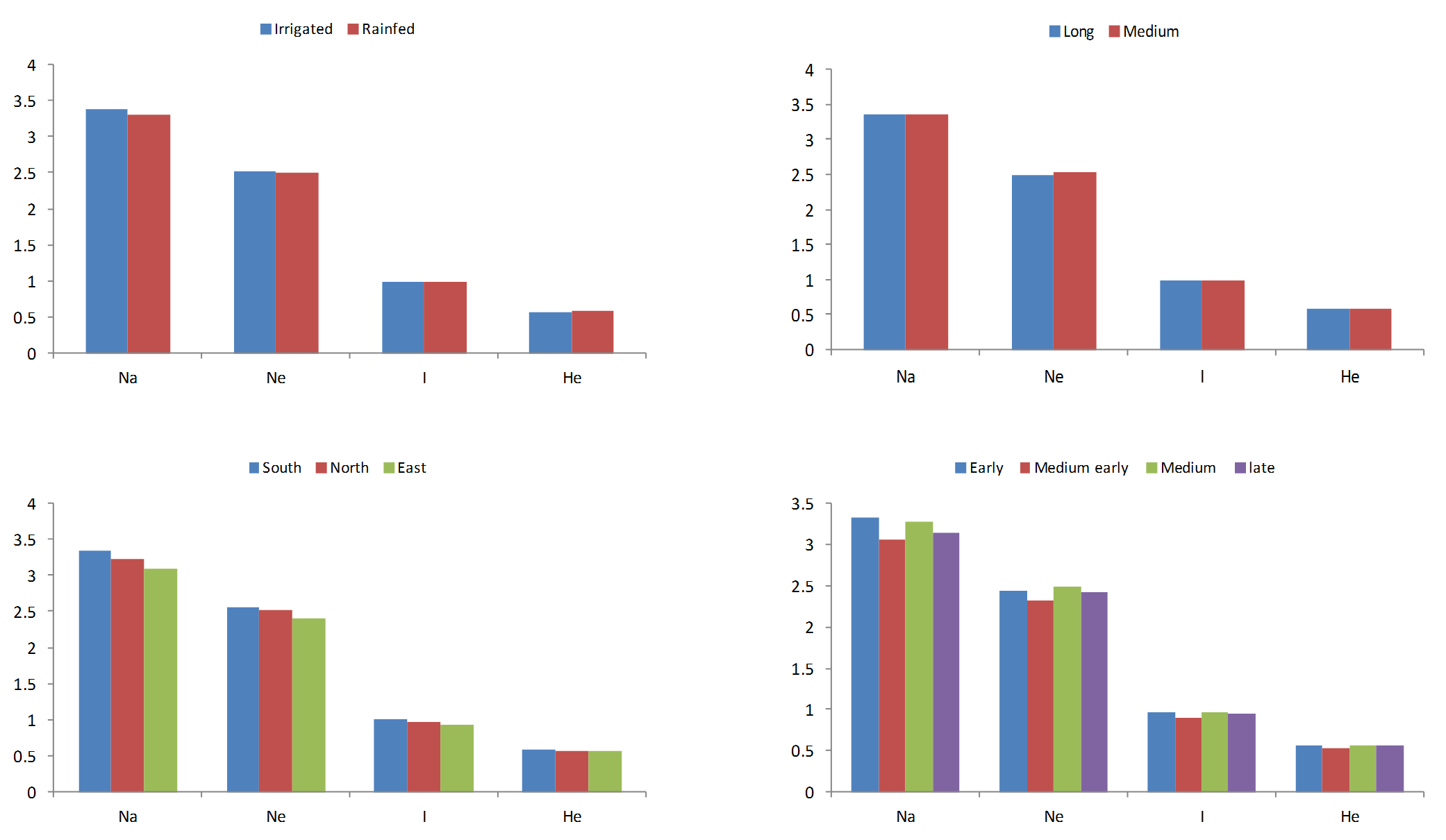

Supplement: Figure S2 — Number of alleles (Na), number of effective alleles (Ne), Shannon index (I) and Nei’s genetic diversity (He) estimated in different groups of rice varieties i.e., region, ecology, grain size and days to 50% floweringwise. (TIF) [file pone.0066197.s002.tif]
