# Supplementary material for: Molecular Genetic Diversity of Major Indian Rice Cultivars over Decadal Periods
Source: PLoS One. 2013 Jun 21;8(6):e66197. doi: 10.1371/journal.pone.0066197 (PMC3689748; doi:10.1371/journal.pone.0066197)
Supplement: Table S2 — Summary statistics of genetic diversity parameters of major Indian rice cultivars. (DOCX) [file pone.0066197.s004.docx]

|  | **Na** | **Rs** | **PIC** | **Ho** | **He** | **Ne** | **I** |
| --- | --- | --- | --- | --- | --- | --- | --- |
| **Year of releasewise** |  |  |  |  |  |  |  |
| Landraces | 2.865 | 1.519596 | 0.8 | 0.1387 | 0.486 | 2.1894 | 0.8206 |
| 1970s | 2.885 | 1.545904 | 0.8 | 0.1582 | 0.520 | 2.2814 | 0.8685 |
| 1980s | 3.231 | 1.567692 | 0.83 | 0.1505 | 0.558 | 2.4854 | 0.9602 |
| 1990s | 3.173 | 1.550558 | 0.843 | 0.1416 | 0.537 | 2.3236 | 0.912 |
| 2000s | 3.308 | 1.560154 | 0.85 | 0.1518 | 0.546 | 2.36 | 0.943 |
| **Longivitywise** |  |  |  |  |  |  |  |
| Landraces | 2.865 | 1.519596 | 0.8 | 0.1387 | 0.486 | 2.1894 | 0.8206 |
| 1970s | 3.000 | 1.549308 | 0.7873 | 0.1631 | 0.527 | 2.3084 | 0.8882 |
| 1980s | 3.250 | 1.575923 | 0.8335 | 0.152 | 0.568 | 2.5201 | 0.9764 |
| 1990s | 3.289 | 1.577654 | 0.8343 | 0.1425 | 0.570 | 2.4776 | 0.9722 |
| 2000s | 3.327 | 1.586635 | 0.8355 | 0.152 | 0.579 | 2.526 | 0.9902 |

Table S2 Summary statistics of genetic diversity parameters of major Indian rice cultivars

Na- Number of alleles; Rs- Allelic richness; PIC-Polymorphism information content; Ho-Observed heterozygosity; He-Expected heterozygosity; Ne-Number of effective alleles; I- Shannon index
