# Supplementary material for: Molecular Genetic Diversity of Major Indian Rice Cultivars over Decadal Periods
Source: PLoS One. 2013 Jun 21;8(6):e66197. doi: 10.1371/journal.pone.0066197 (PMC3689748; doi:10.1371/journal.pone.0066197)
Supplement: Table S3 — Locus by locus AMOVA among populations. (DOCX) [file pone.0066197.s005.docx]

| Locus | d.f. | Va | % variation |
| --- | --- | --- | --- |
| RM562 | 4 | 0.03949 | 10.53597 |
| RM11313 | 4 | 0.06326 | 21.28973 |
| RM11340 | 4 | 0.01973 | 7.66228 |
| RM11356 | 4 | 0.01001 | 3.5421 |
| RM11597 | 4 | 0.03286 | 11.15569 |
| RM12031 | 4 | 0.00331 | 4.22692 |
| RM12253 | 4 | 0.02265 | 7.43088 |
| RM12292 | 4 | 0.01127 | 3.34701 |
| RM12353 | 4 | 0.10321 | 33.78226 |
| RM12548 | 4 | 0.01602 | 5.21772 |
| RM13131 | 4 | 0.0097 | 3.48992 |
| RM13584 | 4 | 0.02617 | 8.06459 |
| RM14270 | 4 | 0.03848 | 11.30745 |
| RM14735 | 4 | 0.01083 | 2.79846 |
| RM14778 | 4 | 0.01229 | 3.83449 |
| RM15004 | 4 | 0.00614 | 1.86921 |
| RM15580 | 4 | 0.00755 | 2.54782 |
| RM16416 | 4 | 0.03096 | 17.24942 |
| RM16577 | 4 | 0.02256 | 7.06747 |
| RM17405 | 4 | 0.03112 | 12.14069 |
| RM17669 | 4 | 0.01901 | 6.38174 |
| RM5693 | 4 | 0.00084 | 0.27456 |
| RM5844 | 4 | 0.01117 | 3.26448 |
| RM5907 | 4 | 0.00338 | 1.38855 |
| RM18384 | 4 | -0.00063 | -0.31942 |
| RM18639 | 4 | 0.01577 | 4.5444 |
| RM19545 | 4 | 0.01093 | 3.73102 |
| RM20037 | 4 | 0.02866 | 8.8708 |
| RM20710 | 4 | 0.02038 | 5.20893 |
| RM21693 | 4 | 0.05267 | 15.33942 |
| RM21941 | 4 | 0.01595 | 5.40443 |
| RM22250 | 4 | 0.01116 | 3.10507 |
| RM22554 | 4 | 0.01181 | 3.18953 |
| RM22565 | 4 | 0.00217 | 0.63414 |
| RM22688 | 4 | 0.00512 | 2.69667 |
| RM22273 | 4 | 0.0309 | 8.91422 |
| RM23017 | 4 | 0.04798 | 17.82604 |
| RM23036 | 4 | 0.01436 | 4.41343 |
| RM23362 | 4 | 0.05825 | 16.88683 |
| RM23741 | 4 | -0.00412 | -1.39737 |
| RM24015 | 4 | 0.00903 | 2.54605 |
| RM24044 | 4 | 0.03191 | 12.43923 |
| RM24260 | 4 | 0.0284 | 8.82012 |
| RM5708 | 4 | 0.02254 | 6.36667 |
| RM8207 | 4 | 0.00037 | 0.10651 |
| RM25262 | 4 | 0.0226 | 9.44091 |
| RM25969 | 4 | 0.02274 | 11.12457 |
| RM26190 | 4 | 0.01128 | 3.77928 |
| RM26632 | 4 | 0.01584 | 5.50279 |
| RM27840 | 4 | 0.01953 | 6.55344 |
| RM28279 | 4 | 0.02122 | 6.75733 |
| RM6965 | 4 | 0.06372 | 22.14986 |

Table S3 Locus by locus AMOVA among populations
