# Supplementary material for: Molecular Genetic Diversity of Major Indian Rice Cultivars over Decadal Periods
Source: PLoS One. 2013 Jun 21;8(6):e66197. doi: 10.1371/journal.pone.0066197 (PMC3689748; doi:10.1371/journal.pone.0066197)
Supplement: Table S4 — Population specific alleles and their corresponding markers. (DOC) [file pone.0066197.s006.doc]

|  |  |  |  |  |  |  |  |  |
| --- | --- | --- | --- | --- | --- | --- | --- | --- |
| Decade | Specific alleles | Markers containing specific allele | | |  |  |  |  |
| Landraces | 6 | RM12031 | RM15004 | RM5844 (170) | RM21693 | RM22250 | RM23362 |  |
| 1970s | 0 |  |  |  |  |  |  |  |
| 1980s | 1 | RM18384 |  |  |  |  |  |  |
| 1990s | 1 | RM5708 |  |  |  |  |  |  |
| 2000s | 2 | RM5844 (190) | RM23741 |  |  |  |  |  |
| **Longevity wise** |  |  |  |  |  |  |  |  |
|  |  |  | |  |  |  |  |  |
| Decade | Specific alleles | Markers containing specific allele | | |  |  |  |  |
| Landraces | 7 | RM12031 | RM15004 | RM5844 (170) | RM21693 | RM22250 | RM23362 | RM8207 |
| 1970s | 0 |  |  |  |  |  |  |  |
| 1980s | 1 | RM18384 |  |  |  |  |  |  |
| 1990s | 1 | RM5708 |  |  |  |  |  |  |
| 2000s | 2 | RM5844 (190) | RM23741 |  |  |  |  |  |

Table S4 Population specific alleles and their corresponding markers

**Year of release wise**
