# Supplementary material for: Molecular Genetic Diversity of Major Indian Rice Cultivars over Decadal Periods
Source: PLoS One. 2013 Jun 21;8(6):e66197. doi: 10.1371/journal.pone.0066197 (PMC3689748; doi:10.1371/journal.pone.0066197)
Supplement: Table S5 — List of genic and non-genic SSRs and their functions. (DOC) [file pone.0066197.s007.doc]

| Table S5 List of genic and non-genic SSRs and their functions |  | **Genic SSRs** |  |  |  |
| --- | --- | --- | --- | --- | --- |
| **S.No.** | **SSR Locus** | **Chromosome** | **Motif** | **Locus name** | **Gene function** |
| 1 | RM562 | 1 | (AAG)13 | Os03g44250 | Hypothetical protein |
| 2 | RM12353 | 2 | (AAT)32 | Os02g02830 | Ubiquitin-conjugating enzyme E2-17 kDa 9, putative, expressed |
| 3 | RM13131 | 2 | (AT)32 | Os02g25010 | Hypothetical protein |
| 4 | RM13584 | 2 | (AATC)5 | Os02g39570 | Acetolactate synthase/ amino acid binding protein, putative, expressed |
| 5 | RM17669 | 4 | (AT)30 | Os04g58770 | Hypothetical protein |
| 6 | RM5844 | 5 | (AAT)20 | Os05g16180 | NB-ARC domain containing protein |
| 7 | RM5907 | 5 | (AAT)19 | Os05g51630 | HYP1, putative, expressed |
| 8 | RM18384 | 5 | (AAG)22 | Os05g25540 | Phytosulfokine receptor precursor, putative, expressed |
| 9 | RM18639 | 5 | (AAT)17 | Os05g33570 - | Pyruvate, phosphate dikinase, chloroplast precursor, putative, expressed |
| 10 | RM20037 | 6 | (AT)38 | Os06g28194 | Phosphoglucomutase/phosphomannomutase family protein, putative, expressed |
| 11 | RM22250 | 8 | (AAT)30 | Os04g02030.1 | Protein coding |
| 12 | RM23036 | 8 | (AGAT)15 | Os08g30480.1 | Clathrin adaptor complex small chain domain containing protein, expressed |
| 13 | RM23362 | 8 | (AAG)19 | Os08g38710.1 | Uncharacterized glycosyltransferase, putative, expressed |
| 14 | RM23741 | 9 | (AAT)28 | Os09g04910.1 | Hypothetical protein |
| 15 | RM24044 | 9 | (AAG)11 | Os09g16458.1 | Pleiotropic drug resistance protein 4, putative, expressed |
| 16 | RM24260 | 9 | (AAT)31 | Os09g23720.1 | Expressed protein |
| 17 | RM25969 | 11 | (AAG)18 | Os11g01630.1 | Retrotransposon protein, putative, Ty3-gypsy subclass |
| 18 | RM28279 | 12 | (AATC)8 | Os12g32374.1 | Histone deacetylase 6, putative, expressed |

**Non-genic** SSRs

| **SSR Locus** | **Chromosome** | **Motif** |
| --- | --- | --- |
| RM11313 | 1 | (AAT)23 |
| RM11340 | 1 | (AT)42 |
| RM11356 | 1 | (AT)31 |
| RM11597 | 1 | (AT)42 |
| RM12031 | 1 | (AG)40 |
| RM12253 | 1 | (AAT)23 |
| RM12292 | 1 | (AT)38 |
| RM12548 | 2 | (AT)46 |
| RM14270 | 3 | (AT)46 |
| RM14735 | 3 | (AT)42 |
| RM14778 | 3 | (AT)37 |
| RM15004 | 3 | (AAT)38 |
| RM15580 | 3 | (AT)50 |
| RM16416 | 4 | (AAT)12 |
| RM16577 | 4 | (AT)29 |
| RM17405 | 4 | (AAT)36 |
| RM5693 | 5 | (AAT)18 |
| RM19545 | 6 | (AAT)21 |
| RM20710 | 6 | (AT)46 |
| RM21693 | 7 | (AT)44 |
| RM21941 | 7 | (AAT)25 |
| RM6965 | 7 | (AAG)15 |
| RM22554 | 8 | (AAT)20 |
| RM22565 | 8 | (ACAT)15 |
| RM22688 | 8 | (AAT)28 |
| RM22273 | 8 | (AT)35 |
| RM23017 | 8 | (AAT)18 |
| RM24015 | 9 | (AGAT)9 |
| RM5708 | 10 | (AAT)22 |
| RM8207 | 10 | (AAG)23 |
| RM25262 | 10 | (AAT)38 |
| RM26190 | 11 | (AGAT)13 |
| RM26632 | 11 | (AAAG)9 |
| RM27840 | 12 | (AAT)37 |
