# Supplementary material for: Molecular Genetic Diversity of Major Indian Rice Cultivars over Decadal Periods
Source: PLoS One. 2013 Jun 21;8(6):e66197. doi: 10.1371/journal.pone.0066197 (PMC3689748; doi:10.1371/journal.pone.0066197)
Supplement: Table S6 — Summary statistics of genetic diversity parameters of Indian rice cultivars using different microsatellite classes. (DOCX) [file pone.0066197.s008.docx]

|  | **Na** | **He** | **Ne** | **I** |
| --- | --- | --- | --- | --- |
| **Genic SSRs** | |  |  |  |
| Landraces | 3 | 0.4635 | 2.1736 | 0.8109 |
| 1970s | 2.8889 | 0.5524 | 2.3957 | 0.9104 |
| 1980s | 3.2778 | 0.5686 | 2.4996 | 0.9819 |
| 1990s | 3.1111 | 0.5436 | 2.2826 | 0.9158 |
| 2000s | 3.3889 | 0.5626 | 2.3775 | 0.9784 |
| **Non-genic SSRs** | |  |  |  |
| Landraces | 2.7941 | 0.4976 | 2.1977 | 0.8257 |
| 1970s | 2.8824 | 0.5029 | 2.2209 | 0.8463 |
| 1980s | 3.2059 | 0.5528 | 2.4779 | 0.9487 |
| 1990s | 3.2059 | 0.5335 | 2.3453 | 0.9106 |
| 2000s | 3.2647 | 0.5367 | 2.352 | 0.9247 |
| **Gene-specific indels (Previous reports)** | | | |  |
| 1960 | 1.824 | 0.4118 | 1.8235 | 0.5708 |
| 1970s | 2.158 | 0.4084 | 1.7818 | 0.6242 |
| 1980s | 2.316 | 0.3981 | 1.7885 | 0.6194 |
| 1990s | 2.211 | 0.2923 | 1.5112 | 0.4843 |
| 2000s | 2.053 | 0.3995 | 1.7408 | 0.5977 |

Table S6 Summary statistics of genetic diversity parameters of Indian rice cultivars using different microsatellite classes

Na- Number of alleles; He-Expected heterozygosity; Ne-Number of effective alleles; I- Shannon index
