# Supplementary material for: Molecular Genetic Diversity of Major Indian Rice Cultivars over Decadal Periods
Source: PLoS One. 2013 Jun 21;8(6):e66197. doi: 10.1371/journal.pone.0066197 (PMC3689748; doi:10.1371/journal.pone.0066197)
Supplement: Table S7 — Summary statistics for genetic diversity parameters of Indian rice varieties classified according to their region, ecology, grain size and days to 50% flowering. (DOCX) [file pone.0066197.s009.docx]

| **Varietal groups** | **Sample size** | **Na** | **Rs** | **PIC** | **Ho** | **He** | **Ne** | **I** | **Private alleles** | **Rare alleles** | **Common alleles** |
| --- | --- | --- | --- | --- | --- | --- | --- | --- | --- | --- | --- |
| **Statewise** |  |  |  |  |  |  |  |  |  |  |  |
| North India | 43 | 3.23 | 3.17 | 0.83 | 0.16 | 0.57 | 2.52 | 0.98 | 4 | 10 | 5 |
| South India | 21 | 3.35 | 3.11 | 0.85 | 0.15 | 0.58 | 2.55 | 1.00 | 3 | 8 | 2 |
| East India | 17 | 3.10 | 3.02 | 0.80 | 0.14 | 0.56 | 2.40 | 0.93 | 0 | 16 | 6 |
| **Ecology** |  |  |  |  |  |  |  |  |  |  |  |
| Irrigated | 41 | 3.38 | 3.31 | 0.84 | 0.16 | 0.57 | 2.52 | 0.99 | 5 | 6 | 3 |
| Rainfed | 31 | 3.31 | 3.26 | 0.83 | 0.14 | 0.58 | 2.50 | 0.98 | 1 | 15 | 3 |
| **Grain size** |  |  |  |  |  |  |  |  |  |  |  |
| Long grain | 49 | 3.37 | 3.23 | 0.84 | 0.15 | 0.57 | 2.50 | 0.99 | 3 | 8 | 1 |
| Medium grain | 31 | 3.37 | 3.27 | 0.84 | 0.15 | 0.58 | 2.54 | 0.99 | 3 | 25 | 2 |
| **Days to 50% flowering** |  |  |  |  |  |  |  |  |  |  |  |
| Early | 32 | 3.33 | 3.09 | 0.84 | 0.15 | 0.56 | 2.44 | 0.96 | 3 | 21 | 2 |
| Medium early | 15 | 3.06 | 2.95 | 0.81 | 0.17 | 0.53 | 2.31 | 0.90 | 0 | 20 | 4 |
| Medium | 18 | 3.27 | 3.09 | 0.83 | 0.13 | 0.57 | 2.49 | 0.97 | 2 | 22 | 3 |
| Late | 22 | 3.13 | 3.04 | 0.84 | 0.15 | 0.56 | 2.42 | 0.96 | 0 | 7 | 2 |

Table S7 Summary statistics for genetic diversity parameters of Indian rice varieties classified according to their region, ecology, grain size and days to 50% flowering

Na- Number of alleles; Rs- Allelic richness; PIC-Polymorphism information content; Ho-Observed heterozygosity; He-Expected heterozygosity; Ne-Number of effective alleles; I- Shannon index
